# On welfare pluralism, social policy and the contribution of sociology: Revisiting Robert Pinker

**DOI:** 10.3389/fsoc.2023.1076750

**Published:** 2023-04-17

**Authors:** John Offer

**Affiliations:** School of Applied Social and Policy Sciences, Ulster University, Coleraine, United Kingdom

**Keywords:** welfare pluralism, social policy, informal care, Titmuss, Robert Pinker, agency, care of older people

## Abstract

On occasion it makes sound sense to undertake a retrospective review of a late colleague's contribution to his or her subject area. This applies to Robert Pinker, Professor of Social Administration at the London School of Economics, who died at the age of 89 in February 2021. Over a long life he made a major impact on working for press freedom and to social work studies, but this article concerns his work on social policy, and particularly on the idea of welfare pluralism, a many-faceted idea the exploration of which powered two pathbreaking books *Social Theory and Social Policy* (1971) and *The Idea of Welfare* (1979). In the twentieth century many states including the United Kingdom had greatly expanded their welfare provisions for their citizens, and, in some, an academic subject area, often called social administration or social policy had grown in response. Pinker started writing in the 1960s, dissatisfied with the conventional approach of Richard Titmuss and others, almost exclusively concerned with the state and welfare. He made the case for a radical rebalance toward including everyday experiences of obligations and how familial informal welfare practices are strengthened, weakened or modified by formal social services. However, ahead of his time, Pinker was arguing for an *enhanced sociological imagination* in the study of social policy and on the very idea of “welfare”. This article has sections reflecting the facets of Pinker's thinking about welfare pluralism, including “social policy's past”, “exchange and stigma”, “taking informal welfare seriously”, “divergent views of altruism”, “comparative studies”, “on a mixture of means to welfare” and “aspects of Pinker's legacy”. The idea of welfare pluralism is now familiar. But Pinker's crucial pioneering role, depth of understanding of the issues and grasp of their intertwining is seldom recalled. This article should help to meet the need for his contribution to be reinserted into the mainstream of sociological thought on welfare, so enriching new research.

## Introduction

The death of Robert Pinker in his ninetieth year on February 2nd 2021 marked the passing of one of the most distinguished and original senior contributors to the understanding of social policy and welfare. While the United Kingdom was an important focus, he was a much-traveled and observant lecturer; while his reputation ensured his books were translated into Dutch, Serbo-Croat, Japanese and Korea. His hallmark was the adoption of **a** clearly sociologically informed frame of reference in the focus on welfare. His chief books, *Social Theory and Social Policy* of 1971 and *The Idea of Welfare* of 1979, around which this article is itself structured, were pathbreaking. For students and for their teachers they opened new windows on to that field. While Richard Titmuss had recommended that, alongside the statutory social services, consideration should be given to the impacts of taxation and occupation-based benefits such as pension schemes on the distribution of income, Bob Pinker was planting his foot much more boldly outside the frame of orthodox social policy studies of the day. He asked how in everyday life do ordinary people, with their own values and priorities, and across the generations, go about enhancing their own welfare and that of their families, and also of nearby non-relatives. These were questions as much about *the sociology of welfare* as about social policy, and they were then largely unposed questions whether in sociology or social policy studies. The idea of welfare pluralism and ancillary concepts such as the co-production of welfare are now familiar. But Pinker's crucial pioneering role, depth of understanding of the issues and grasp of their intertwining is seldom recalled. This article should help to meet the need for his contribution to be reinserted into the mainstream, so enriching new research.

From that springboard he went on to develop ideas of familial altruism and its conditionality, of links between dependence, stigma and welfare outcomes, and of the nature and scope of welfare pluralism (as opposed to the “institutional model” of welfare provision, namely welfare unitarism). He gave full acknowledgment of the pivotal contributions of the health and personal social services to the “states of welfare” which people achieved, but there were other means to welfare as well as the “welfare state”, and those means gave space to diverse values, traditions and outcomes in the process.

Pinker's distinctive approach to understanding welfare and policy in social life contrasted with that being cultivated by Richard Titmuss and others, in fact a prevailing normative set of presumptions shared by many social science scholars of the day, commonly associated with Fabian socialism. As Julian Le Grand later expressed matters (but while acknowledging Pinker as an earlier author of the criticism), this was inclined to treat ordinary people as “pawns”, subject to the “superior” decision-making powers of policy specialists having responsibility for the delivery of services by the state.

Bob Pinker began his academic career as a research officer in Richard Titmuss's Department of Social Administration at the London School of Economics and Political Science in the late 1950s. As a researcher and higher degree student under Brian Abel-Smith and Peter Townsend, his research contributed to Abel-Smith's *The Hospitals 1800–1948* (and to his own *English Hospital Statistics, 1861–1938* of 1966) (Pinker, 1966) and Townsend's book on accommodation for older people *The Last Refuge*.^1^ Successive academic appointments followed at Goldsmiths College, Chelsea College and then LSE, from where he retired in 1996 as Professor of Social Administration.

## Social theory and social policy

Bob Pinker wrote *Social Theory and Social Policy* in the form of separate though closely related essays. His aim was not to provide an all-purpose textbook, but to refresh the study of welfare and social policy through addressing a series of specific conceptual and theoretical problems. Information may date but good ideas do not. Looking back from the perspective of today the book remains striking for the number of high-caliber and stimulating big ideas which, as they were identified, were begging for more attention, but were not receiving it.

The book was published in 1971. Pinker began with a review of the subject area of social administration, as social policy studies was then called. Social policy was often taught under the umbrella of sociology, but in practice theory tended to be normatively framed toward extending and improving the “welfare state”. Pinker shows that “theory” was largely made up of variations on normative “residual” and “institutional” models of welfare ideas and practice (see Titmuss, 1974). Still often used today as models, originating it seems in the USA (Wilensky and Lebeaux, 1965), the models built in a state-centered starting-point, though with the residual model it is state-centered in a reluctant rather than a positive sense. The subject had little use for specifically “classic” sociological theory as it then was. Pinker discussed Spencer, Durkheim, Marx and Weber, giving different and cogent reasons why each failed to find favor in the emerging subject area. Spencer frowned on state involvement, Durkheim had relatively little to say on collective social policy, Weber had doubts about the consequences of social planning, and Marx had no confidence that the state could meets needs whilst capitalism dominated.

Pinker, however, accurately identified key basic deficiencies in the knowledge needed to underpin more strongly the production of policy thought and practice, addressed in neither of the models nor more widely in social policy studies. We have, he observes, “little reliable evidence about attitudes to social services or expectations of welfare policy and workers” (Pinker, 1971/2022, p. 104); we lack adequate explanations of “why individuals define their needs as they do, and why these definitions so often appear to be at variance with those of the social scientists” (Pinker, 1971/2022, p. 106); and we know “almost nothing about the reasons for which citizens use services as they do, or about what attitudes lead them to feel deterred or encouraged in the search for assistance” (Pinker, 1971/2022, p. 202). Failure to “take account of the experience and subjective reality of the everyday life of ordinary people” is the underlying cause (Pinker, 1971/2022, p. 104). As he was writing, newer and theoretically more applicable approaches were filtering from sociology to social policy studies.^2^ Indeed, there have been many incremental gains in understanding from studies owing much to these approaches (e.g., Carrier and Kendall, 1973, 1977). Yet it is still the case that these matters are not lying where they should be, at the heart of the study of social policy.

When Pinker wrote, prescriptive visions of the good life, from both left and, less often, from the right, ruled the roost (Pinker, 1971/2022, p. 110), rather than sociologically-inclined and methodologically robust studies of “current levels of satisfaction and discontent” among ordinary people, which documented the reasons why they themselves “hold the range of attitudes and expectations they do”. Then and now, these are surely crucial areas of the knowledge needed if we are to understand in plausible and persuasive terms the objectives and outcomes of social policy in the context of a free and democratic society, yet they are available relatively infrequently. Recently, Beresford referred to “a ‘social administrative' model for understanding and analyzing public policy. This was typified by Fabian social policy where the political was underplayed and policymaking … best left to academic and other ‘experts”' (Beresford, 2019, p. 2). It was exactly that limitation with the analysis of social policy as it stood of which Pinker despaired back in 1971 (see Exley, 2019): he was certainly *not* mounting a “rearguard defense of ‘orthodox' social administration” at all, though he was depicted as such in (Lee and Raban, 1988, p. 5).

### Relative deprivation

Pinker himself in fact singles out as “impressive” the sociological work of W.G. Runciman in his *Relative Deprivation and Social Justice* (Runciman, 1966) with its original focus on the subjective realities of poverty, though it was while also regretting that it was largely ignored, as remains too frequently the case today.

Pinker also found encouragement in the “capability approach” to understanding and identifying injustice associated with the work of Sen. In *The Idea of Justice* (Sen, 2009), Sen argues that in life we do things, we have freedom to choose and not only to do things for *our own* wellbeing. The actual capabilities people have, the power to do things and the things they realize, come with a sense of responsibility for what we do. Moreover, the actual capabilities people have, and the things people want to do, brings us to further issues “that turn out to be quite central to the analysis on justice in the world” (Sen, 2009, p. 19**)**.

As against the “utility-based or resource-based lines of thinking”, the capability approach takes an approach to social actors as active agents, and judges individual advantage by “a person's capability to do things he or she has reason to value. A person's advantage in terms of opportunities is judged lower than that of another if she has less capability—less real opportunity—to achieve those things that she reason to value” (Sen, 2009, p. 231). In social policy studies itself there is some sympathy for the approach (e.g., Hick, 2012), however, there is probably more resistance to it especially where agency, unlike structural features in society, is looked at askance (Dean, 2009).

Recently, “outcome-based” accountability measures have become a popular approach to policymaking and monitoring. However, may they well not dig deep enough methodologically to yield the fine-grained consciousnesses of experientially based lives which Pinker, and Sen, have sought (Birrell and Gray, 2018).

### Exchange and stigma

A further influential theme of *Social Theory and Social Policy* covers exchange and stigma. In *Commitment to Welfare* Richard Titmuss urged a distinction, which become frequently cited, between gifts or unilateral transfers which characterized the “social market” and exchange or bilateral transfers which characterized the “economic market” (Titmuss, 1968, p. 22). Pinker criticized robustly this distinction as unduly speculative. Did the community share the belief that the state provided the values that were most likely to minimize stigma? Pinker asked for relevant research, not least to show whether, in everyday life, there is a distinction made between the relative powers of “givers” and “receivers” of services, in which the values of the economic market are also reflected in social welfare systems.

Titmuss himself selected voluntary blood donations as a case study to provide a test of where the “social” begins and where the “economic” ends. To this end, Titmuss reached out to social anthropology, in the shape of *The Gift (Essai sur le don)* by Mauss (1990), originally published in 1925. For Titmuss, that study gives us a lesson in how we can provide and extend “opportunities for altruism in opposition to the possessive egoism of the market place” (Titmuss, 1970, p. 13). However, the anthropologist Mary Douglas tellingly believed that Mauss would have disapproved of the use Titmuss made of his work: Mauss would have said “Nonsense!” to Titmuss's idea “that the archetypal pure-gift relationship is the anonymous gift of blood, as if there could be an anonymous relationship. Even the idea of a pure gift is a contradiction” (Douglas, 1990: x; see also Fontaine, 2002: 424). Hart has similarly observed that “Mauss's chief ethical conclusion is that the attempt to create a free market for private contracts is utopian and just as unrealizable as its antithesis, a collective based solely on altruism” (Hart, 2014, p. 41). Mauss himself, then, had rejected the Titmussian binary opposition for which Titmuss uses Mauss as a source. The “pure” models of selfish vs. generous economic action distract from the complex interplay between our individuality and belonging in subtle ways to others. Indeed, in *The Idea of Welfare* Pinker gives prominence to the plurality of avenues, from families to markets and to the state itself, by which people may choose to pursue their own and their family's welfare and wellbeing, and that of others.

Pinker concluded it was uncertain that Titmuss's and the general Fabian belief that universal social services would minimize felt stigma and the low take-up of benefits as compared with services administered under a selective, means-tested regime. Once again, the paramount need was to gain reliable knowledge about the social reality of felt obligation in everyday life, or its absence, and to enable that knowledge to be so disseminated as to permeate the pores of policymaking and delivery at all levels (Pinker commented further on Titmuss in Ch 5 in Offer and Pinker, 2017, p. 93–112).

Given as current facts of social life that conditional lines are drawn between concern for others and self-interest, whether strong or weak, the exercise of stigmatization is, as Pinker observed, “a highly sophisticated form of violence in so far as it is rarely associated with physical threats or attack. It can best be compared to those forms of psychological torture in which the victim is broken psychically and physically but left to all outward appearances unmarked” (Pinker, 1971/2022, p. 175). Naturally, stimulation in some form of Durkheimian “moral education” can be encouraged, with any ensuing modifications in values and action being duly registered in the practical “sociology of morals” as Pinker well understood (Pinker, 1971/2022, p. 180); but the world as it is experienced needs to be distinguished from what one would prefer it to be, whether for better or worse. Robert Walker's *The Shame of Poverty* (Walker, 2014) has more recently explored a similar landscape within a comparative perspective; Pinker should be recognized as a pathfinder for Walker's study.

However, it is 43 years after *Social Theory and Social Policy first* appeared, with its criticisms of the lack of focus on the experiences of everyday life in social policy studies that Pinker was then advancing. And those well-grounded criticisms have been just outlined again here. It is, therefore, astonishing with the passage of all those years, yet perfectly justifiable, that Walker still it feels it is necessary to underline that “people experiencing poverty must be the experts on their own condition”. “Studying poverty and making policy”, adds Walker, “in the absence of such insights, while commonplace, is logically indefensible and likely to result in distortion and to policies that are ineffectual and counterproductive”. Naturally, it may be that people experiencing poverty may need further information; without it “they may not be able fully to contextualize what they know or to isolate individual and structural causes and correlates” (Walker, 2014, p. 182–83). But this level of analysis is one that must be developed in partnership and dialogue with those experts on their experiences of their everyday lives, not misread as a substitute for experiences themselves.

### Social policy's past

A notable feature of his work how Pinker traces the origins of social policy studies in the nineteenth century, with the prevailing political and economic ideas of those origins being set within the wider context of the times. Nascent policy-making including the use of research as “hard” evidence to fuel the drive for often rival schemes of social reform and the overarching commitment to tackling “social problems”, by lobbying for fresh legislation and extended administrative responsibilities. Pinker had conducted research into the English Poor Law, publishing *English Hospital Statistics* in 1966, and accordingly he shared important insights into Poor Law reform in the 1830s, and into the development of a general hospital service from 1867 within its auspices (Pinker, 1966), first in London and then beyond–in England and Wales by 1891 “3 in every 4 hospital beds” were provided by Poor Law institutions (Pinker, 1971/2022, p. 72).

As a generic and complex institution before the “welfare state”, the Poor Law richly merited this level of attention, still denied it in many social policy studies. A degree of local variety in provision between Poor Law Unions could allow an opportunistic failure to comply with the guidance of the central controlling administration, often to the benefit of local interests and the local poor themselves. The Poor Law structure itself, with its schools and outdoor and indoor medical provision, and similar relief to older people, meant that the “stigma” of receiving poor relief became largely an issue in the twentieth century for the able-bodied unemployed and their families.

Inevitably this leads us on into the hugely significant Royal Commission on the Poor Law (set up in 1905; issuing its majority and minority reports in 1909). The Royal Commission of 1909 was divided on the way forward, for the Fabian minority, led by Beatrice Webb, the Poor Law should be broken up, with specific needs allocated to separate and specialized services staffed by experts, while the majority, led by Helen Bosanquet recommended rebuilding it as Public Assistance, with a strong input from the voluntary sector.

Pinker shrewdly remarked that the minority report was “autocratic in its prescriptions and insensitive to the realities of everyday life for ordinary people” (Pinker, 1971/2022, p. 83), whereas the majority was decidedly more sympathetic in that regard. Here lay a conflict to surface again in Pinker's own experience. Although the immediate impacts on policy of both reports were negligible, comparing and contrasting the reports still provides vivid insights into the complex and frequently still-enduring texture of the theoretical and ideological variables as applied to the ends and means of achieving welfare accompanied by democratic values.

When Unions and the Poor Law ceased to relieve the unemployed in 1937, once a new central body the Unemployment Assistance Board of 1935 finally assumed the task, the prime motive was to assert strong central control over finance. This step removed the “problem” of the leniency of some Unions toward the relief of their unemployed; it was *not* primarily about freeing claimants from the allegedly unpopular Poor Law but imposing centralized and tighter financial controls! (Gilbert, 1970, p. 188).

## The idea of welfare

It is will soon be a quarter of a century since Pinker's book *The Idea of Welfare* was originally published. Since the 1970s I have followed what he has written with a keen interest and more recently, I have been fortunate enough to often share his company while we were working on jointly editing a volume of his essays on welfare pluralism. Sadly, the COVID pandemic intervened before his death but many enjoyable telephone calls and emails continued. In my own work I know that I have returned to *The Idea of Welfare* again and again ever since it was originally published in 1979: over the span of four decades its happy knack of identifying pivotal questions about how to make sense of “welfare” in theory and practice remains undimmed.

However, for many first-time readers of this book the landscape of social policy and politics in the United Kingdom of 40 years ago is likely to be remote, so two key events around the time of its earlier publication should be noted. First, the year 1979 was only 6 years after the UK, and Ireland, had joined what we now call the EU: in the UK that event had gained further approval in a referendum in June, 1975. Second, in 1979 itself, there was a general election in the United Kingdom on May 3, giving the Conservative Party an overall majority with Margaret Thatcher as Prime Minister. James Callaghan, the defeated Labor Prime Minister, had “never had a reliable majority in Parliament” according to Denis Healey, his Chancellor of the Exchequer (Healey, 1989, p. 448), and Callaghan's Government had suffered an economy with pronounced rates of inflation (around 16% in 1976 and 1977) and high-profile labor disquiet, the dubbed the “winter of discontent”. The social policy specialist Michael Hill has sensibly cautioned us against falling into “the trap of believing that in 1979 a wicked witch emerged to cast a spell over the British welfare state”. It was just too simplistic to add “ism” to “the end of Thatcher's name” (Hill, 1993, p. 122). Nevertheless, after 1979 there were significant changes to come in thinking about the roles of social workers, as discussed in the Barclay Report (1982) *Social Workers: Their Role and Tasks*, and the funding and delivery of “community care”, following the Griffiths Report *Community Care: Agenda for Action* (Griffiths, 1988). These were two areas of particular interest to Pinker: he was a member of the group preparing the Barclay Report for the government, and he wrote many articles from the 1980s and later which separated the rhetoric from realities in discussion of the mixed economy of welfare and social care (see Offer and Pinker, 2017).

However, there are more specific events and controversies in the practice and study of social policy in advance of 1979 that help explain the richness of original features and the enduring stimulation of *The Idea of Welfare*. The Report of the Wolfenden Committee entitled *The Future of Voluntary Organizations* in 1978 was one such significant event. Some discussion of that Report also provides a natural link into the exploration of the fundamental and instructive contrasts in the analytical priorities of thought in Richard Titmuss and Bob Pinker.

### Taking informal welfare seriously

*The Future of Voluntary Organizations* (the Report of the Wolfenden Committee, 1978) was made possible by the support of the Joseph Rowntree Trust and the Carnegie United Kingdom Trust. The Committee reviewed the role and functions of voluntary organizations for the next quarter century. It assumed that “the advent of what is comprehensively known as ‘The Welfare State”' (Wolfenden Committee, 1978, p. 9) had made timely a review. As well as also considering relationships with the state and commercial providers for meeting need, the Wolfenden report felt that it very significant indeed that a further “system” of meeting needed required urgent and sustained consideration, what it called “the informal network of support provided by family, friends and neighbors” (Wolfenden Committee, 1978, p. 15). While this kind of support had achieved very limited attention in an earlier official Report to the government on social work, the *Report of the Committee Local Authority and Allied Personal Social Services* of 1968 (the Seebohm Report, 1968), it was the Wolfenden Report which inaugurated the process whereby the topic became incorporated as a central focus in “official” recommendations for the future direction of social policy in the UK. However, the Labor Government's response to Wolfenden, *The Government and the Voluntary Sector: A Consultative Document* (Home Office and Voluntary Services Unit, 1978), was of interest in that it seemed bewildered about the meaning of the ‘informal system': the only reference to the topic is the narrow and awkward one of “volunteers caring for dependent relatives” (Home Office and Voluntary Services Unit, 1978, p. 32), not, as might be expected, “*family members* caring for dependent relatives”.

In 1973 Pinker had published his Lecture “The Welfare State”, arising from his preparatory work on *The Idea of Welfare*, in which he remarks that “[t]he way in which ordinary citizens define and seek to enhance their own state of welfare merits as much attention as the ways in which academics define the welfare state” (Pinker, 1973, p. 3). In November 1977, he wrote a Report to the Research Initiatives Board of the (then) Social Sciences Research Council, *Research Priorities in the Personal Social Services*, recommending the encouragement of studies into the “changing framework of assumptions about the relationship between community care and residential care, the role of the informal care systems and the balance between the statutory, voluntary and private care sectors” (Pinker, 1977a, p. 35–36). Pinker was already familiar with pioneering books and articles featuring studies of informal care such as Mayer and Timms (1970), Bayley (1973), Glastonbury et al. (1973), Topliss (1975), and Glampson et al. (1977). Other valuable sources then placing a spotlight on informal care include Collins and Pancoast (1976), Abrams (1978) (on Abrams's research see also Bulmer, 1986), and Robinson (1978). All this research shared the central belief in the sound sociological approach that understanding the social relations themselves as interpreted by the actors involved was pivotal, as opposed to applying from the start a narrowly administrative and normatively-driven view as to what would be “useful” for policy and practice.

At the time*, The Idea of Welfare* was unique as a synoptic and sociologically informed study of welfare, and as focused moreover on controversial concepts and principles at stake in the degree of attention given to matters pertaining to informal care. Of course, Pinker was already primed to recognize the shortcomings which afflicted the focus of earlier traditions of social policy research. The lesson of Wolfenden was that a book was overdue to draw more explicit and wider attention to the new research area, then on the verge of mushrooming (on which see Parker, 1990). The neglect of informal care as a form of welfare and its interfaces with statutory and voluntary social services was reprehensible enough, but it had become united with the lamentable neglect of other linked and wider key matters on which a well-grounded knowledge of everyday social life and welfare interactions needed to depend:

we need to understand more fully the ways in which and the extent to which changes occur among ordinary people with regard to their definitions of felt obligation and entitlement, because such shifts in opinion and belief provide the groundswell of support for official policies as well as the counter-policies which are the neglected dimension of social welfare studies (Pinker, 1979/2019, p. 42).

In this book and in earlier *Social Theory and Social Policy* arguably *the* heart of his message was the need to listen to the voices and see the actions of ordinary people as active agents.

It was becoming clear, therefore, that ordinary people are going about the putting together the best welfare and wellbeing outcomes they can for themselves and the members of their families as they regard it, and sometimes other people as well. It must then be an oversight that Deacon and Mann describe Pinker as “almost wholly” neglecting “agency” (Deacon and Mann, 1999, p. 415). A no less questionable claim about social policy and administration in general has been made: “in the 1960s and 1970s”, Welshman reported, “the notion of human agency was ignored” (Welshman, 2004, p. 226). Further comments on the place of agency are made in the later section “On a Mixture of Means to Welfare”.

### Divergent views of altruism

Richard Titmuss had died in 1973. In 1977 Pinker observed that “few scholars have so dominated the development of an academic subject over so long a period of time as did Richard Titmuss”. The subject area was social policy and administration; these days, of course, usually known as social policy. Titmuss was “one of those rare thinkers who are able to shift the whole focus of debate in a field of study and thereby open up entirely new areas of intellectual enquiry” (Pinker, 1977b, p. vii). While Pinker had studied and researched with Titmuss (and with Peter Townsend and Brian Abel-Smith) at the London School of Economics, by the 1970s he had become Lewisham Professor of Social Administration, Goldsmiths and Bedford Colleges London 1972–74, and then Professor of Social Studies, Chelsea College London 1974–78. Then he returned to LSE, becoming Professor of Social Work Studies 1978-93 and Professor of Social Administration 1993–96.

In 2006, when reflecting on Titmuss's influential book of 1970, *The Gift Relationship: From Human Blood to Social Policy*, Pinker remarked that both his own earlier book *Social Theory and Social Policy* of 1971 and his *The Idea of Welfare*

were written largely as critiques of Titmuss's analysis of the moral dynamics of welfare institutions, the uncompromising distinction he drew between egoism and altruism, and the unitary model of social policy on which his analysis was based. I thought that his ideal of social welfare as “a major integrated institution”—were it ever to be realized—would impose nothing less than an intellectual and normative straightjacket on the diversity of policy ends and means that ought to characterize a free society. I preferred the idea of a pluralist mixed economy of welfare which took more account of the ambiguities and paradoxes of human nature and gave more opportunities for us all to pursue what The Book of Common Prayer describes as “the devices and desires of our own hearts” (Pinker, in Offer and Pinker, 2017, p. 108).

Pinker's critical engagement with Titmuss is at the heart of *The Idea of Welfare*. His criticism takes neither the Marx-indebted pathways of Ginsburg's *Class, Capital and Social Policy* (Ginsburg, 1979) and Gough's *The Political Economy of the Welfare State* (Gough, 1979), nor Hayek's uncompromising liberal individualism of *Individuals and Social Order* (Hayek, 1976). If a normative welfare unitarism characterized Titmuss's outlook, as Pinker has judged it, a model of welfare pluralism, in the first place descriptive, was foremost in Pinker's own mind. For Pinker, much of Titmuss's writing was “charged with the intensity of his moral commitment to a vision of collectivist social progress” (Pinker, in Offer and Pinker, 2017, p. 107). Early in *The Idea of Welfare*, Pinker argues that the task in the study of social welfare

is not merely to make judgements about the anomalies and paradoxes of our social welfare system but to describe as fairly as possible the scope and limits of social welfare and to account for the phenomena which we are describing. We must go beyond the construction of models which set out ideological positions rather than explaining actual situations and events, and models presented in terms which appear to be simply descriptive, but which are in fact highly value-laden (Pinker, 1979/2019, p. 5).

In a similar vein, Deacon described Titmuss as “first and foremost a moralist” (Deacon, 2002, p. 197). The historian Jose Harris was more exact; Titmuss's “social philosophy … was full of the muffled resonances of the Idealist discourse of the Edwardian age” (Harris, 1999, p. 59–60). This was passed on to Titmuss through R.H. Tawney from philosophers such as T.H. Green as Oxford. The State and its arms possessed a holistic grasp of moral insight and virtue, and welfare, above that was available to ordinary citizens.^3^

Pinker did not share that view, instead drawing attention to “conditional altruism”, which refers to, say, in the familial and thus informal context, of negotiating how best to use its resources to advance the welfare and wellbeing of its members. Family decisions are likely to take the relative needs, merits and deserts of the members in to account.^4^ Pinker argues that with reference to conditional altruism, and against Titmuss, that analyses relating to welfare matters which discuss sentiments in terms of “egoism” and “altruism” as polar opposites in social life, do not take adequate account “of the subtle interplay of loyalties which characterize people”s notions of welfare obligation and entitlement” (Pinker, 1979/2019, p. 10). These ideas are also explored with particular reference to “reciprocity” in Bulmer (1986, p. 103–117).

Pinker made the further the analytical point that a sound understanding of social policy needs to know the rules of entitlement and obligation that pertain in *formal interactions* of, say, social workers and service users, and also the *everyday and informal version* of such “rules” of entitlement and obligation as interpreted in actual familial and communal settings. The capacity for conflict but also opportunistic cooperation between agile actors moving across these different sets of “rules” or terrains means that without it we lack a necessary insight into what makes an adequate “typology of social welfare” (Pinker, 1979/2019, p. 43).^5^

The formal/informal distinction poses a real difficulty in Titmuss: what is intended to be descriptive can become one-dimensional, and what is intended to be normative or prescriptive, can become too inflexible. For Pinker, the acceptability of particular social policies in social life derives “not only from their instrumental effectiveness but from the sense of meaning and significance they have for the citizenry” (Pinker, 1979/2019, p. 43). His interests were not the binary opposite of those of Titmuss but the different nuances were substantial and prescient, most visibly over the direction of sociology and in particular in the area of moral and generative agency within everyday life.

However, it is notable that Pinker sharply distinguished his own research-led position from any kind of “populism”, being skeptical of any alleged populist “consensus” as furnishing an acceptable basis for the content of social policy and aims of social welfare (Pinker, 1984). In 2019, as populist movements grew apace, something he not imagined in 1979, Pinker worried that their supporters and leaders are “so strongly disposed to end up at the polar ends of the left-right political spectrum and then claim their extremist views on all the big political issues, represent the ‘general will' of ordinary people” (Pinker, 2019).

### Comparative studies

The lack of intellectual depth in comparative studies in the study of welfare concerned Pinker, and part of *The Idea of Welfare* is devoted to comparative developments in the UK, the USA and Russia, adopting the wide typology of social welfare he had summarized. The new focus is on the distinctive cultural expectations, social histories and political traditions which are represented, and on the nature of the differing experiences of ordinary people as a range of social and political changes were unfolding, with a particular emphasis on their perceptions of the welfare-related implications upon them. While it might be relatively straightforward to quantify basic differences (and similarities) between the places involved, Pinker was tackling the task of showing how divergent concepts and theories of “welfare” itself were associated with divergent historical, cultural and social situations, at home as well as abroad (compare Higgins, 1981, p. 163–67, Ashford, 1986; see also Baldock, 1999).

Two examples may be highlighted. On historical change in Russia, Pinker observes, “the question of land reform dominated all other issues of social welfare”. Modern forms of social service were of relatively marginal importance in what was achieved by the government and in what the mass of the population expected: “as late as 1917 80 per cent of the Russian population were still peasants for whom the ultimate form of welfare was the possession of land” (Pinker, 1979/2019, p. 142). The other example, evidenced beyond the discussion of Russia, is that, before the emergence of modern welfare states, “emigration was a traditional means by which men tried to provide for their welfare on their own terms” (Pinker, 1979/2019, p. 230). There are connections between these two examples: they both remain pressing issues today (Pinker returned to these topics in “Citizenship, civil war and welfare: the making of modern Ireland”, in *Twenty-first Century Society*, vol. 1, no 1, 23-38, 2006, reprinted in Offer and Pinker, 2017, 171–186, with an accompanying “Afterthought”).

Both examples open up the salutary point that there is much to learn “about the welfare practices of ordinary people in the area of social life which are unregulated by formal policies and expert opinions, We can add a new dimension to our knowledge if we give more attention to the times when the range of such formal regulation was far more limited than it is today” (Pinker, 1979/2019, p. 197).

### On the sociology of welfare and a mixture of means to welfare

An interesting theme in *The Idea of Welfare* is Pinker's disavowal of Titmuss's rather bald dichotomy between “the social market” (allocation of resources by the state) and “the economic market”, arguing they are in social realty inextricably linked. The policy analyst and economist Martin Knapp noted Pinker's demonstration of how Titmuss had “generated a deal of confusion” with that “false dichotomy”, although Knapp's own and optimistic belief that the dichotomy “seems to have been largely dispelled” was probably premature (Knapp, 1984, p. 10, compare Pinker, 1979/2019, p. 247). When associated with a “welfare pluralist” or mixed economy of welfare perspective, the two “markets” are seen as *co-producers* of welfare, with the important participation of informal care and voluntary organizations alongside.^6^

Julian Le Grand, the Richard Titmuss Professor of Social Policy at the London School of Economics since 1993, had come to a similar outlook to Pinker in the conclusion in the 2006 edition of his *Motivation, Agency, and Public Policy*. In that version he included a Postscript in which was a substantial extract from Pinker's essay “From gift relationships to quasi-markets: an odyssey along the policy paths of altruism and egoism” (of 2006, reprinted in Offer and Pinker, 2017, p. 209–223). Le Grand now recognized (Le Grand, 2006: 208):

Until I read Robert Pinker's essay, I had not realized the extent to which his two seminal works—*Social Theory and Social Policy* (1971) and *The Idea of Welfare* (1979)—had prefigured mine, and I am glad to have the opportunity to acknowledge this. He criticized simplistic notions of altruism and egoism, pointing out that most people are driven by a combination of altruistic and egoistic motives and that this fact must be taken into account in any development of social policy. He emphasized that the extent of the sacrifice involved in an altruistic act is an important test of the scope and limits of altruism as a moral motivator. He was an early advocate of pluralistic systems of welfare—at a time when it was deeply unfashionable to do so. He argued that the most authentic rights that we have are those of the market place.

As has been emphasized already, in these achievements Pinker imported a strong sociological dimension. From the outside social work, the study of social policy and sociology often blur into one. But they are not. Bob Pinker saw the urgency of bringing much more of the sociological imagination into the study of social policy in the 1970s, and there were some studies taking that view (e.g., Warham, 1973). It seemed by the end of century, though, it was argued that a greater *rapprochement* was still desirable (Offer, 1991, 1999a). Since welfare relations are always intertwined with social relations (as Morris, 2020 has recently emphasized), that remains the case.

## Aspects of Pinker's legacy

Pinker makes a pragmatic further suggestion that it is within a welfare pluralist setting the most likely and best hopes of flexible and responsive approaches to improve the prospects for welfare and wellbeing for UK citizens, in what is itself, in many respects, a pluralist and complex society, with both linear and non-linear dynamics of change with which to contend. In this context, attention must be drawn to the fact that Pinker's work, dating from the 1970s, in has had a striking but unacknowledged resonance in recent and important theoretical developments in aspects of the sociology of welfare, as presented, for example, in Graham Room's *Complexity, Institutions and Public Policy: Agile Decision-Making in a Turbulent World*, 2011, and *Agile Actors on Complex Terrains: Transformative Realism and Public Policy* (2016). Indeed, when Pinker observes that the bonds of kinship and family form “one of the most potent sources of what might be called the ‘counter-policies' of social welfare” (Pinker, 1979/2019, p. 41) we witness a pre-echo of what Room has referred to as range of “institutional terrains”.

Actors in these terrains are increasingly “agile”, being well-informed and “wise” to the world. Most actors are involved in several terrains at once, say families, neighbors, voluntary bodies, in employment (part-time or full-time), using corner shops, supermarkets and food banks, and being claimants of social security payments. The basic premise has become that we now live, in the 21st Century, in a world of increasingly agile and informed choice- making agents. According to the information those agents access and the calculations they reach, they are potential users or non-users of a range of services from policy-providers and professionals, and also from informal sources.

There are commonly trade-offs between the goals pursued on one terrain and those pursued on another; and there may be scope for cooperation or conflict, not just within individual terrains but across them. Just as important, the outcome of interactions on one terrain may affect the resources which each actor can then bring to the struggle on another; or, indeed whether they can even gain access (Room, 2016, p. 86). Thus, as circumstances alter, the householder, as “agile institutional entrepreneur”, renegotiates the “complex web of formal and informal social affiliations”, including any given openings for employment in which they are enmeshed (Room, 2011, p. 257). The contributions that may be offered by family, friends and neighbors come into the picture, and if a job is available the question becomes whether or not taking the job is likely to dovetail adequately with the children's childcare needs.

In “uncertain and foggy landscapes”, or environments if we prefer the word, the relationships which professionals need to observe with governments and regulators must figure as much in “adaptive walks” as with their relationships to other active agents, including individuals, families and communities. And all these social actors are likely to be seeking positional advantage (Room, 2016, p. 100–101).

As has been alluded to already, Pinker suggests that the family “has always been an object of suspicion among social reformers” (Pinker, 1979/2019, p. 38). Families and individual family members, and others, are more likely to be active agents that passive observers of their lives. Pinker made an explicit call to develop within the study of welfare relations, informal and formal, what he described as a “sociology of morals”. Among its keys tasks would be to study “the extent to which the values and assumptions which are implicit in social legislation support, weaken or modify the moral beliefs and practices of ordinary people” (Pinker, 1974, p. 8–9; others too, including Le Grand, arrived at the same conclusion).

However, and in an importantly novel and substantive way, this matter has very recently been followed through in a strand of sociological research from Australia, which responds strongly, though apparently unknowingly, to the spirit of Pinker's call. This work takes up the challenge of “telling sociological stories of hope” in circumstances of hardship, but not by “romanticizing everyday struggle” among families and individuals receiving welfare benefits (Mitchell, 2022, p. 487). There is a distinction to be made here between being an “adept responder” as a receiver of benefits on the one hand, and pursuing wider goals to have “a liveable life in accounts of getting by” on the other (Mitchell, 2022, p. 491). As Mitchell has remarked, a sociological approach is more likely to bring out the difference than policy-oriented research, which can submerge generative aspects of agency under a “dominant concern with resilience and resistance”:

This makes sense in policy-oriented research that seeks to understand how people respond to threats to their welfare and how the architecture of informal and formal support affects their capacity to do so. However, it crowds out a view of how means of getting by may also be more than that; even channels of satisfaction, desire, and accomplishment (Mitchell, 2022, p. 491).

Observing this distinction can thus serve as a corrective to the tendency of research which can otherwise eclipse what Mitchell shows us to be “the wider struggle for dignity and worth in the face of ‘welfare identities' that engender shame” (Mitchell, 2020, p. 236).

The work of both Room and Mitchell can be slotted comfortably into a wider tradition of significant sociological investigations, stemming from Pinker's efforts to highlight the area of moral and generative agency involving everyday life and welfare, to which it forms a fitting legacy.^7^ To recognize connections like these retrospectively is itself worthwhile.

However, the biggest bonus would come in transforming the connections into action in order to advance future research. Our understanding of welfare relations as social relations would expand more quickly once the connections made were joined up together and sifted. The reach of the varied fruits of the research imaginations across the generations would be realized and their impact enlarged, with new questions generated as a direct consequence.

## Conclusion

Many years have passed since the books by Pinker first appeared, pushing informal care and carers into public gaze. As it happens, in the second decade of the 21st Century, the United Kingdom has yet to deliver a comprehensive financial package in support of the older people with long-term needs for care, while the proportion older people in the population continues to grow. It was suggested recently that “where, in the 1980s, informal carers were just beginning to make their voice known, their contribution to the mosaic of care is now established and assumed”: the building of partnerships with carers is an important challenge for services which “knit together ‘caring solutions' which are neither exploitative, neglectful nor dismissive of the user/carer's own way of doing things” (Holloway and Lymbery, 2007: 377). What the eventual resolution will be remains unknown, but it is now very probable indeed that when a financial package does appear, those voices will he heard. Pinker's challenge to the status quo in the 70s will have not been in vain.

It is important to mention before this review concludes the friendship between the sociologist Tom Marshall and Bob Pinker. Pinker only occasionally referred to Marshall in the *The Idea of Welfare* (in the final Chapter) but Marshall's influence is clear in the book. Marshall and Pinker share a preference for middle range theories and skepticism toward the siren songs of unfettered capitalism and communism. Pinker subsequently edited an important book on Marshall (1981) and significantly developed aspects of Marshall's work on citizenship (in Offer and Pinker, 2017). Indeed, it is possible that Marshall was on Pinker's mind when he pitted ideas of welfare pluralism against judging the forms of social organization solely by reference to the two paradigms of capitalism and communism:

this makes us voluntary prisoners of two historical modes of thought, both of which embody highly deterministic theories of social development. Capitalism does not necessarily collapse or suffer irreparable damage if the free play of market forces is modified. Neither does it inevitably give way to socialism when the time and circumstances are ripe. It is perfectly reasonable to argue that capitalism will develop in ways which we are not able to anticipate or predict; it has done so in the past (1979, p. 236).

Pinker died in February 2021, and *Social Theory and Social Policy* with its manifold insights was first published over fifty years ago; in 2029 *The Idea of Welfare* will also pass that mark. He observed in 1971 that ‘racial bigots in Alabama and Russian bullies in Prague' were threatening human wellbeing. Normative theory had to be checked by the role of reason: in a subtler way, “the sociologist who wilfully confuses normative theory with old-fashioned ideology damages rather than enhances the aims of social welfare and justice:” (Pinker, 1971/2022, p. 134). If he were still alive he would describe the current anguish in Ukraine as springing from the same roots.

## Data availability statement

The original contributions presented in the study are included in the article/supplementary material, further inquiries can be directed to the corresponding author.

## Author contributions

The author confirms being the sole contributor of this work and has approved it for publication.

## Footnotes

^1^Pinker's research work on residential care for older people for Peter Townsend's book *The Last Refuge* is discussed in Johnson et al., 2010, p. 7–8).

^2^In broad terms there were insights gained from phenomenology and symbolic interaction. Pinker's reading of *The Social Construction of Reality* by Berger and Luckmann (1967), first published in 1966 was an influence on his thought.

^3^Idealist social thought and welfare is discussed further with reference to the case of Titmuss in Offer (1999b, 2006).

^4^This concept of “conditional altruism” should treated as a separate topic from “welfare conditionality”, although there is possibly some shared ground. “Welfare conditionality” is a principle concerned to make eligibility for specified welfare services and benefits delivered by government conditional to meeting duties to behave “responsibly”.

^5^This concept of “conditional altruism” should treated as a separate topic from “welfare conditionality”, although there is possibly some shared ground. “Welfare conditionality” is a principle concerned to make eligibility for specified welfare services and benefits delivered by government conditional to meeting duties to behave “responsibly”.

^6^Another concept not familiar when Pinker was writing in the 1970s was “personalization”, and a note on it and informal care will be helpful here. “Personalization” is a recent and contested concept within social care services. In 2012 in *Caring for our Future* the government stated: “Personalization is about giving people choice and control over their lives, and ensuring that care and support responds to people's needs and what they want to achieve. It is central to enabling people to lead active, independent and connected lives” (Department of Health, 2012, p. 54). It is also specifically connected “with the devolution of budgets down to the individual or a nominated budget-holder” (Needham, 2015, p. 357). Glasby has remarked that some see personalization as “promoting greater citizenship for disabled people, and trying to ensure greater independent living”. However, others see this as a part of a “neoliberal agenda designed to undermine traditional public sector services and values, transfer responsibility from the state to the individual and ‘dress up' unfair and draconian cuts as a more positive policy reform” (Glasby, 2015, p. 85). “Personalization” is thus producing divergent estimations of its significance in welfare policy, from the positive of enhancing subjective well-being to the negative of viewing people as “atomized subjects of an inexorable neoliberal capitalism” (Williams, 2015, p. 104). However, it should be stressed that there is no *necessary* association between these “negative” forms of “personalization”, sometimes linked to a “neoliberal” agenda, and roles for informal care and social care services in welfare pluralism.

^7^As is discussed in the earlier sections on “relative deprivation” and “exchange and stigma”, Pinker's work also covered feelings of stigma, shame and frustrated desires in social life in the context of welfare relations.
